# Trichilemmal Carcinoma in a Patient With Oculocutaneous Albinism

**DOI:** 10.7759/cureus.81477

**Published:** 2025-03-30

**Authors:** Ty Theriot, Jonathan M Joseph, Lacey Falgout, Christopher Haas

**Affiliations:** 1 Department of Dermatology, Louisiana State University Health Sciences Center (LSUHSC) School of Medicine, New Orleans, USA

**Keywords:** adnexal, immunohistochemistry, nonmelanoma skin cancer, oculocutaneous albinism, trichilemmal carcinoma

## Abstract

Trichilemmal carcinoma (TC) is a rare adnexal neoplasm derived from the outer sheath of the hair follicle. Despite its aggressive histological features, TC typically exhibits low-grade clinical behavior, with local recurrences being more common than metastasis. This case report describes a 47-year-old Hispanic female with oculocutaneous albinism and a history of numerous nonmelanoma skin cancers (NMSCs) who presented with an erythematous papule on her forearm. Histopathology revealed TC characterized by basaloid keratinocytes with clear cytoplasm and peripheral palisading and a high proliferation rate confirmed by Ki-67. Surgical excision with 1.0 cm margins was performed, and final histopathology showed no residual tumor. Concurrently, the patient was diagnosed with metastatic squamous cell carcinoma (SCC) and started on pembrolizumab. This case highlights the increased susceptibility of individuals with oculocutaneous albinism to multiple and varied skin malignancies, stressing the need for vigilant skin surveillance and a broad differential diagnosis in this population. The patient's history underscores the importance of early detection and intervention to manage and mitigate the risk of advanced skin cancers.

## Introduction

Trichilemmal carcinoma (TC) is a rare adnexal neoplasm derived from the outer sheath of the hair follicle. It is a rare condition, with the largest group of cases submitted with 103 patients in a literature review. The pathophysiology of the disease is unclear; however, factors such as ultraviolet radiation, immunosuppression, skin trauma, and genetic diseases like xeroderma pigmentosum and Cowden syndrome have been shown to contribute [1]. Lesions are typically solitary and may present as nodules, plaques, or ulcerated lesions [2]. The characteristic histopathology demonstrates lobules of atypical keratinocytes with glycogen-rich clear cell change, often displaying a pattern of peripheral palisading and a high mitotic rate [3]. Immunohistochemistry is often positive for cytokeratin CK1, 10, 14, 17, and 19 and is negative for the carcinoembryonic antigen (CEA), but there have been late positive results occasionally reported [4,5]. They are also negative for S-100 antigen and cytokines 7, 8, 15, 16, and 18 [6]. While there is no consensus on treatment, surgical excision with at least 1 cm margins has served as safe and effective [7].

Despite its aggressive histological features, the clinical behavior of TC is typically low-grade, with local recurrences being more common than metastasis. However, it is noteworthy to mention that the potential for metastasis exists, especially with delayed diagnosis and treatment. There are a few cases in the literature where metastasis to the lymph nodes is present. One patient was immunosuppressed with prednisone, cyclosporine, and mycophenolate mofetil due to kidney transplantation [8,9].

Oculocutaneous albinism (OCA) is a group of inherited disorders characterized by a generalized reduction in melanin pigment in the eyes, skin, and hair [10]. Individuals with OCA often have sun sensitivity, leading to a predisposition to sunburn and the development of skin cancer. Sun protection is of paramount importance, but despite best efforts, patients often develop numerous nonmelanoma skin cancers (NMSCs) throughout their lifetime [11].

## Case presentation

**Figure 1:**
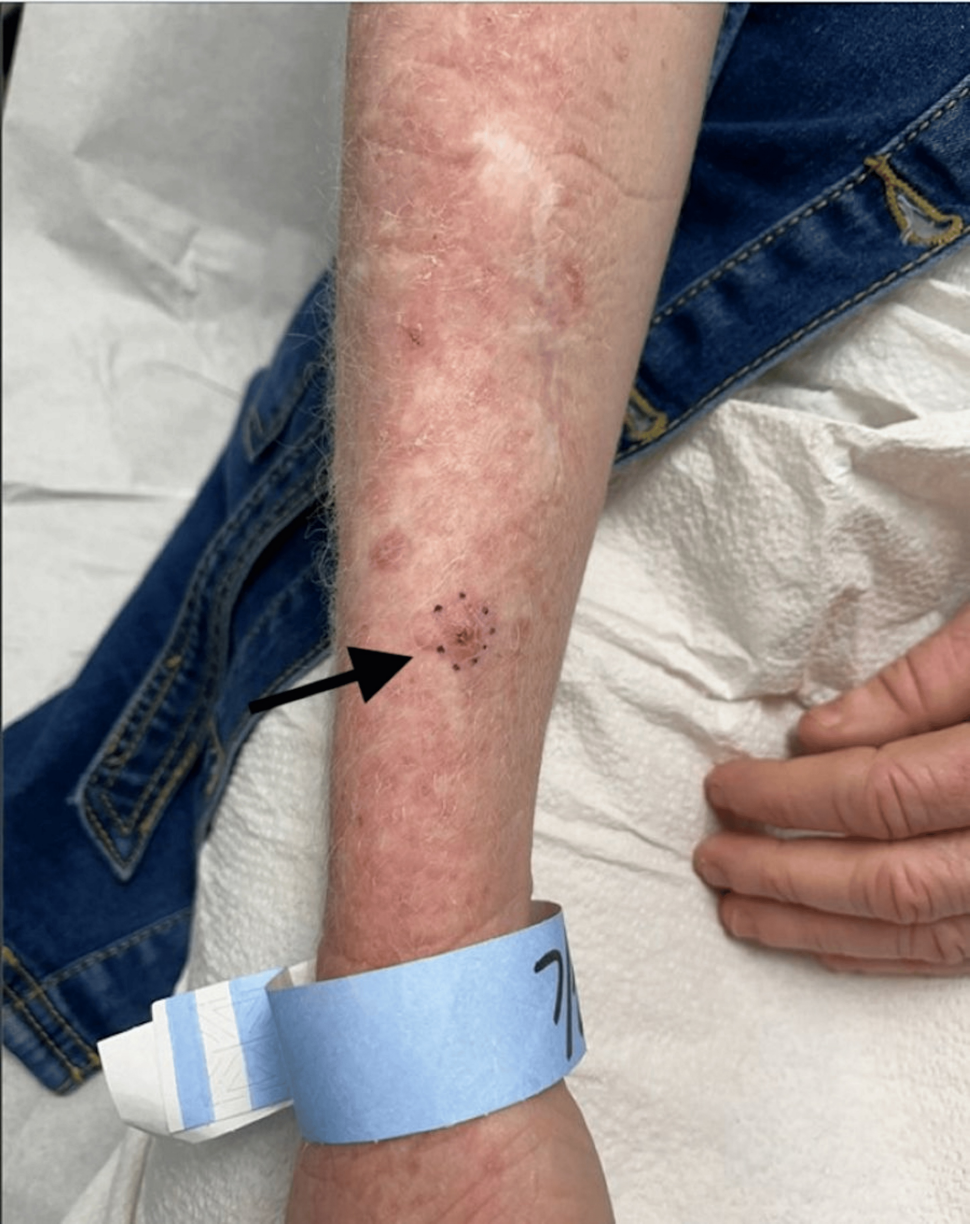
Clinical photograph of the right distal medial forearm with a minimally erythematous papule with central ulceration and hemorrhagic crusting (indicated by black arrow) along with scattered erythematous scaly papules.

 A 47-year-old female with a past medical history of OCA and numerous NMSCs returned to the clinic to re-establish care six months after multiple biopsies were performed, resulting in a nodular basal cell carcinoma (BCC) and an invasive squamous cell carcinoma (SCC). An examination of the right distal medial forearm revealed a minimally erythematous papule with central ulceration and hemorrhagic crusting along with scattered erythematous scaly papules (Figure 1). A shave biopsy was performed.

**Figure 2:**
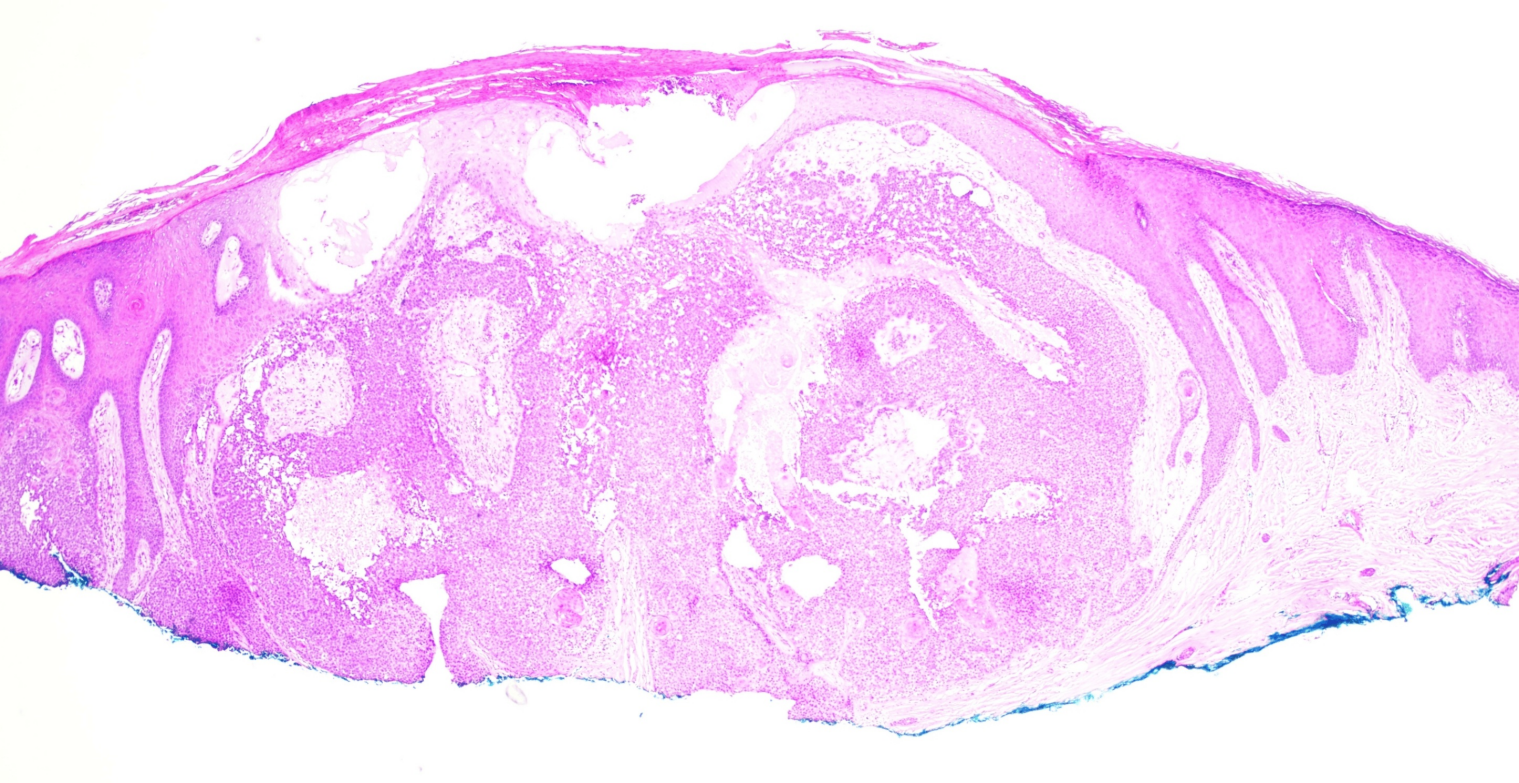
H&E stain at 2× magnification showing a well-circumscribed nodule in the dermis. H&E: hematoxylin and eosin

**Figure 3:**
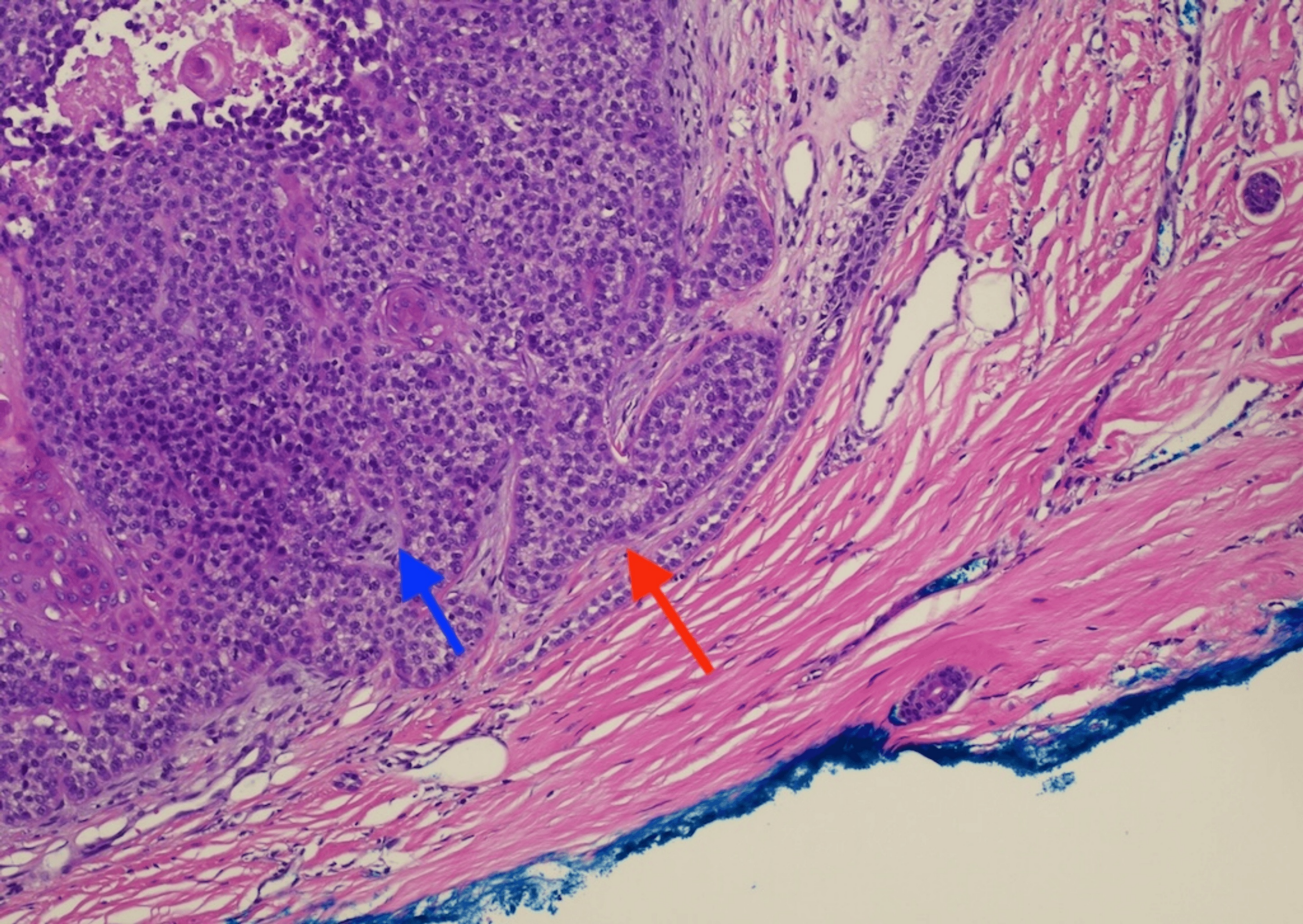
H&E-stained section at 10× magnification showing a proliferation of basaloid keratinocytes in the dermis. Peripheral palisading is indicated by the red arrow, and clear cytoplasm is indicated by the blue arrow. A peripheral scar can also be observed at the margin. H&E: hematoxylin and eosin

**Figure 4:**
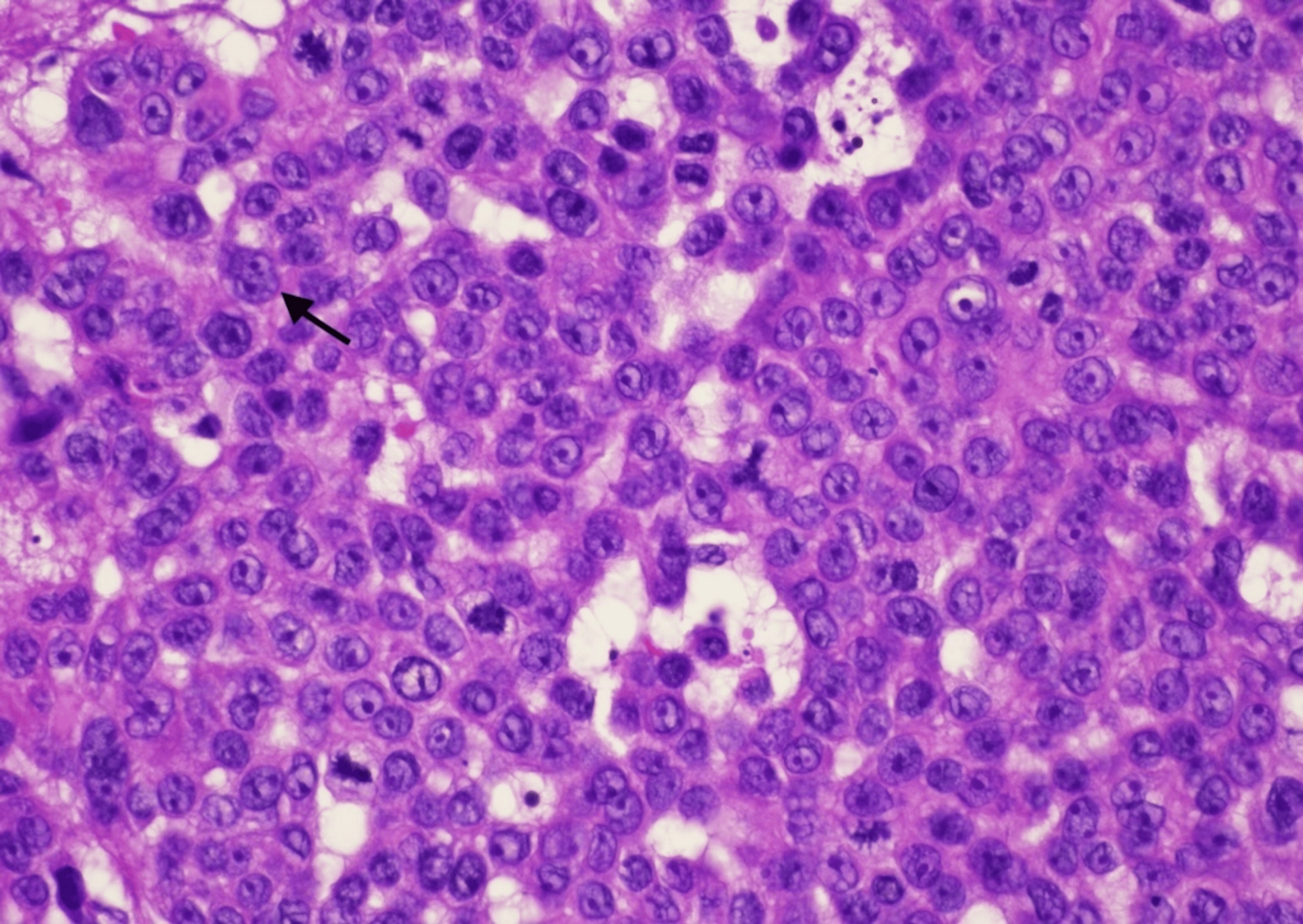
H&E-stained section at 100× magnification showing basaloid keratinocytes with clear cytoplasm. Brisk, abnormal mitotic activity is observed, with a representative mitotic figure indicated by the black arrow. H&E: hematoxylin and eosin

**Figure 5:**
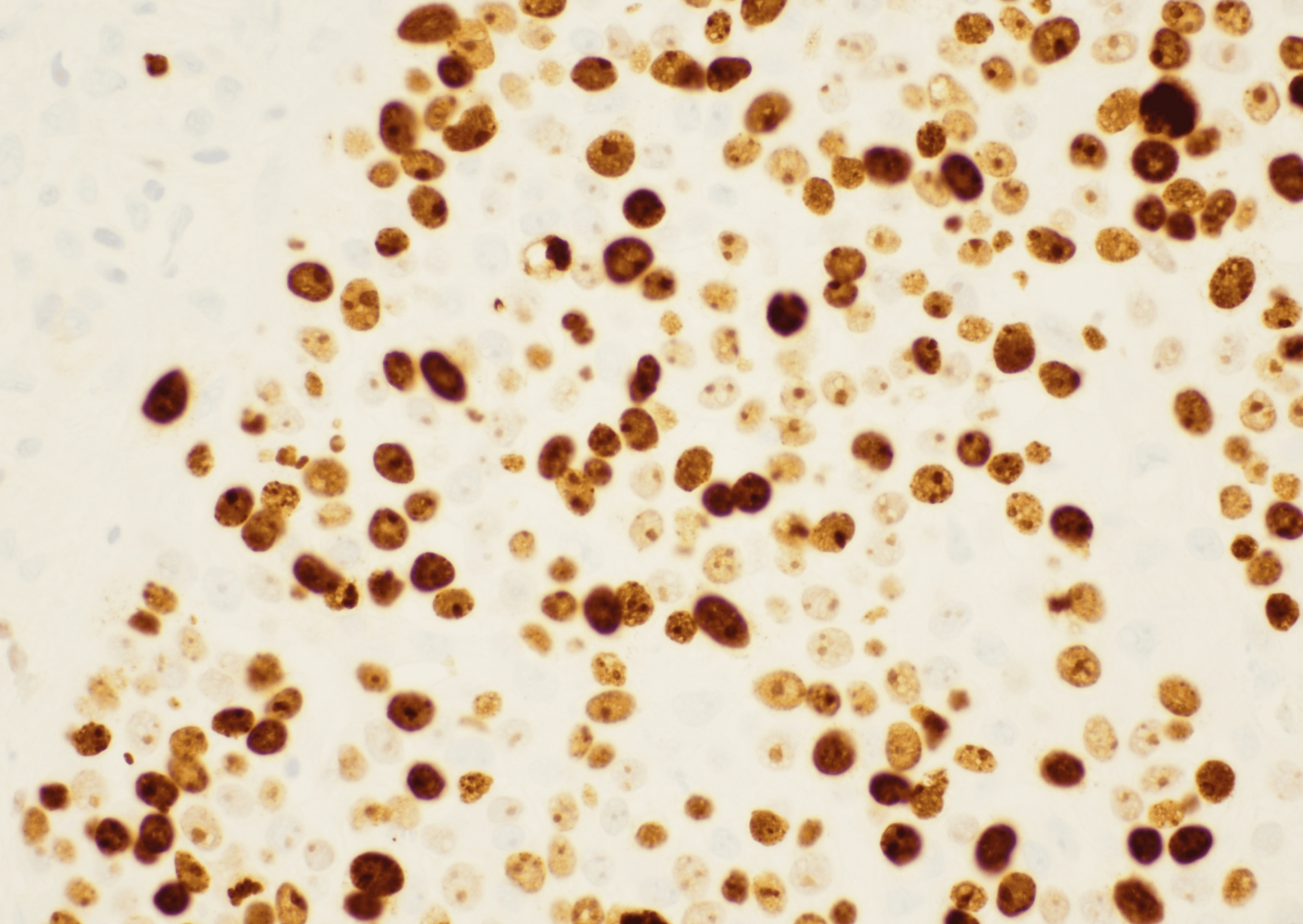
Ki-67 immunohistochemical stain at 100× magnification demonstrating a high proliferation rate (approximately 50–60%). Positive Ki-67 staining is evident as brown nuclear staining within the cells, indicating active cell division.

Histopathology revealed the proliferation of basaloid keratinocytes forming a nodule in the dermis with clear cytoplasm and peripheral palisading. Brisk and abnormal tripolar mitotic activity is identified. Ki-67 shows a high proliferation rate (50-60%), consistent with TC (Figures 2-5).

The patient returned to the clinic one month following the biopsy and was set up for surgical excision. The patient underwent successful excision of the TC with 1.0 cm margins. Final pathology showed no residual tumor. Of note, during this process, the patient was diagnosed with metastatic SCC with presumed skin as the primary source. Subsequently, she was followed up with oncology for treatment with pembrolizumab. She followed up with ophthalmology for OCA, foveal hypoplasia, pendular nystagmus, and bilateral cataracts.

## Discussion

Histopathologic mimickers of TC include squamous cell carcinoma with clear cells (SCC-C), clear cell SCC (associated with Bowen's disease), and proliferating trichilemmal tumor (PTT). Although the broader differential diagnosis for TC also encompasses other cutaneous adnexal tumors and skin cancers (e.g., BCC and sebaceous carcinoma), SCC with clear cell differentiation can be particularly challenging to distinguish from TC histologically [2]. SCC-C often shows intracytoplasmic glycogen and foci of trichilemmal keratinization but lacks consistent immunohistochemical evidence of outer root sheath differentiation (a hallmark of TC) and typically does not express markers such as CD34, CK17, and NGFR/p75 [3]. SCC-C, often associated with Bowen's disease, also features clear cells but lacks the trichilemmal keratinization and glycogen content seen in TC; moreover, it does not show immunohistochemical evidence of trichilemmal differentiation (e.g., CD34) [12]. PTT, by contrast, is typically a well-circumscribed, subepidermal lesion with abrupt keratinization and hyperplastic squamous epithelium, and its malignant variants exhibit high mitotic rates, severe nuclear pleomorphism, and tissue invasion [13]. PTTs may express CD34 and calretinin, which can help distinguish them from other neoplasms [14]. Additionally, clear cell hidradenoma and metastatic clear cell carcinoma remain important considerations, as they can present with similar histopathological features.

Immunohistochemical staining is crucial for the diagnosis of TC. TC typically shows positivity for CD34, which is a marker of outer root sheath differentiation. Periodic acid-Schiff (PAS) staining is also useful, as it highlights the glycogen-rich cytoplasm of the tumor cells. In contrast, TC is usually negative for epithelial membrane antigen (EMA), which helps differentiate it from other clear cell neoplasms [15]. Additional markers such as p63 and cytokeratin 5/6 can be used to support the diagnosis.

Genetic analysis of TC has revealed several mutations that are also common in other skin cancers. *TP53* mutations (e.g., p.Arg213*, p.Arg249Trp, and p.Arg248Gln) are frequently observed and are associated with a more aggressive clinical course. Other genetic alterations include TACC3-FGFR3 and ROS1-GOPC fusions, *NF1*-truncating mutations, *NRAS* mutations, *TOP1* amplification, and *PTEN* deletions. These findings suggest that the molecular pathogenesis of TC may overlap with that of other skin cancers, providing potential targets for therapy [16].

While surgical excision is often favored, there have been reported cases of success with topical 5% imiquimod cream [17] and even the use of pembrolizumab in a patient with distant metastasis [18]. There is currently no standard in follow-up guidelines for patients after they have been treated. This patient has follow-up appointments every two to three months due to the frequency with which she develops skin cancers.

In summary, the differential diagnosis of TC includes SCC, BCC, and sebaceous carcinoma, among others. Immunohistochemistry typically shows CD34 positivity and EMA negativity. Genetically, TC often harbors *TP53* mutations and other alterations common in skin cancers, which may influence its clinical behavior and treatment options.

## Conclusions

This patient's history of multiple, distinct skin malignancies, including BCC, SCC, and now TC, underscores the need for heightened vigilance in individuals with oculocutaneous albinism. OCA confers a well-known predisposition to skin cancer due to reduced melanin protection; however, this case highlights that those malignancies may be not only more frequent but also varied, encompassing both common and rare tumor types. While current screening recommendations emphasize regular skin examinations and strict photoprotection, the emergence of multiple aggressive or rare neoplasms in a single OCA patient suggests that our existing guidelines could be insufficient for a subset of high-risk individuals. Given the pronounced risk, more frequent dermatologic evaluations, or at least earlier referral to specialty clinics, might be warranted for OCA patients, especially those with a history of multiple NMSCs. The typical "every 6-12 months" skin check for high-risk patients could be adjusted to even shorter intervals, such as three to six or, in this patient's case, two to three in those with repeated occurrences of NMSCs or poor follow-up adherence. Additionally, clinicians might consider more proactive measures, such as routine dermoscopy by experienced providers, early biopsy of suspicious lesions, and patient education on the importance of prompt follow-up if concerning changes appear.

In the presented case, the patient's history of OCA has invariably contributed to her vulnerability to various skin malignancies. The presence of nodular BCC and a later diagnosis of metastatic SCC emphasize the complexity and heightened risk faced by this demographic. The diagnosis of TC in this setting underscores the necessity for meticulous skin surveillance in patients with OCA and emphasizes the importance of maintaining a broad differential diagnosis for this demographic.
